# Applying data mining techniques to improve diagnosis in neonatal jaundice

**DOI:** 10.1186/1472-6947-12-143

**Published:** 2012-12-07

**Authors:** Duarte Ferreira, Abílio Oliveira, Alberto Freitas

**Affiliations:** 1 Centro Hospitalar Tâmega e Sousa, EPE, Lugar do Tapadinho, Penafiel, 4564-007, Portugal; 2 Department of Health Information and Decision Sciences, Faculty of Medicine, University of Porto, Porto, Portugal; 3 CINTESIS - Center for Research in Health Technologies and Information Systems, Porto, Portugal

**Keywords:** Data mining, Classification and prediction, Neonatal hyperbilirubinemia, Prognosis

## Abstract

**Background:**

Hyperbilirubinemia is emerging as an increasingly common problem in newborns due to a decreasing hospital length of stay after birth. Jaundice is the most common disease of the newborn and although being benign in most cases it can lead to severe neurological consequences if poorly evaluated. In different areas of medicine, data mining has contributed to improve the results obtained with other methodologies.

Hence, the aim of this study was to improve the diagnosis of neonatal jaundice with the application of data mining techniques.

**Methods:**

This study followed the different phases of the Cross Industry Standard Process for Data Mining model as its methodology.

This observational study was performed at the Obstetrics Department of a central hospital (Centro Hospitalar Tâmega e Sousa – EPE), from February to March of 2011. A total of 227 healthy newborn infants with 35 or more weeks of gestation were enrolled in the study. Over 70 variables were collected and analyzed. Also, transcutaneous bilirubin levels were measured from birth to hospital discharge with maximum time intervals of 8 hours between measurements, using a noninvasive bilirubinometer.

Different attribute subsets were used to train and test classification models using algorithms included in Weka data mining software, such as decision trees (J48) and neural networks (multilayer perceptron). The accuracy results were compared with the traditional methods for prediction of hyperbilirubinemia.

**Results:**

The application of different classification algorithms to the collected data allowed predicting subsequent hyperbilirubinemia with high accuracy. In particular, at 24 hours of life of newborns, the accuracy for the prediction of hyperbilirubinemia was 89%. The best results were obtained using the following algorithms: naive Bayes, multilayer perceptron and simple logistic.

**Conclusions:**

The findings of our study sustain that, new approaches, such as data mining, may support medical decision, contributing to improve diagnosis in neonatal jaundice.

## Background

### Neonatal jaundice

Neonatal jaundice is the most common clinical manifestation of newborns [1-3]. Hyperbilirubinemia, the cause of jaundice, appears in approximately 60% of the newborns at term and almost in all preterm neonates, with prevalence greater than 80% [4,5].

In the vast majority of newborns, jaundice is a benign condition. However, an incorrect or delayed diagnosis may put newborns at risk of developing kernicterus [6,7].

Kernicterus is the chronic form of bilirubin encephalopathy and occurs when the deposition of bilirubin in the brain causes irreversible damage [7,8].

The correct identification of newborns at risk of developing severe hyperbilirubinemia and kernicterus is essential for early treatment. Therefore, preventing the newborn from toxic bilirubin levels, especially for their immature central nervous system, has become a main concern for pediatricians [8,9].

Assessing the risk of neonatal jaundice is currently done with the support of specific nomograms that take into account the age of the newborns, the serum or transcutaneous bilirubin levels and associated risk factors [10]. Bhutani’s nomogram is the most widespread and it is also suggested by the guidelines published by AAP and NICE [4,11].

Despite the use of different methodologies to assess the risk of developing neonatal hyperbilirubinemia, several studies pointed out a growing resurgence of bilirubin encephalopathy and kernicterus, identifying the need to improve diagnosis [12,13].

When predicting bilirubinemia, the isolated use of risk factors is identified as the most poor in terms of predictive ability [14]. In another sense, the evaluations of serum and transcutaneous bilirubin in the first day of life of the newborn have shown a significant correlation with the subsequent development of hyperbilirubinemia [15,16]. However, this correlation is even more significant when the evaluation of measurements of serum or transcutaneous bilirubin are combined with the risk factors, especially when the bilirubin levels are high [1,3,16].

Table 1 presents a comparative analysis between the different predictive methods, according to the outcome and predictive accuracy.

**Table 1:** Comparison of the accuracy of traditional risk assessment strategies (adapted from Keren & Bhutani, 2007)

**Prediction model**	**Predictive outcome**	**AUC**	**95% CI**
CRF			
Chou, 2003	Indication for phototherapy	0.69	not reported
Chou, 2003	TSB > 20 mg/dl;	0.79	not reported
Keren, 2005	TSB > 95th percentile	0.71	(0.66-0.76)
Newman, 2005	TSB > 20 mg/dl	0.69	not reported
Newman, 2005	TSB > 25 mg/dl	0.83	(0.77-0.89)
Pre-discharge TSB			
Keren, 2005	TSB > 95th percentile	0.83	(0.80-0.86)
Newman, 2005	TSB > 20 mg/dl	0.79	(0.77-0.81)
Newman, 2005	TSB > 25 mg/dl	0.83	(0.77-0.89)
Combination of TSB and CRF			
Newman, 2005	TSB > 20 mg/dl	0.86	(0.84-0.88)

AUC – Area under the receiving-operator characteristic curve; CI – Confidence interval; CRF – Clinical risk factors; TSB – Total serum bilirubin.

The predictive outcome – severe hyperbilirubinemia – was defined differently in the presented studies of different strategies for risk assessment. Thus, this definition can affect many important factors found with the different models and also the predictive accuracy of the model [17].

### Data mining

Data mining is one of the newest areas of computer science that uses various statistical techniques, databases, artificial intelligence and pattern recognition (one of the areas of machine learning). The basis of the methodologies of data mining is its ability to find patterns and relationships within large quantities of data that can enable the construction of models that meet the task of assigning the class label at unlabeled cases, the combination of statistical methods and artificial intelligence to the management of databases [18,19].

Data mining techniques have thus successfully been applied in a variety of forecasting tasks [20]. By identifying hidden patterns, data mining can get information that allows a new perspective on certain diseases and to find knowledge that can foster more research in several areas of medicine. The high degree of accuracy of developed models is a good example of data mining's contribution to medicine [21].

In many areas of medicine, data mining has proven to be a huge added value by contributing with new discoveries and improving the results obtained with other methodologies [20].

Thus, the application of data mining techniques can be an excellent way to improve the diagnosis of neonatal jaundice, contributing to the reduction in cases of newborns whose misjudgment of the risk of the development of hyperbilirubinemia can put them in danger. To our knowledge, no other study used data mining techniques to improve the diagnosis of neonatal jaundice.

Hence, the purpose of this study is to improve the diagnosis of neonatal jaundice with the application of data mining techniques.

## Methods

This study followed the different phases of the Cross Industry Standard Process for Data Mining model as its methodology [22].

### Business understanding

Different recent studies point out the need to improve the diagnosis of neonatal jaundice to prevent severe hyperbilirubinemia and kernicterus. Hence, it is important to explore new methodologies, such as data mining, that can provide better results than the traditional methods.

After examining the different data mining tools, the software WEKA version 3.6, was chosen mainly because of its characteristics: it is a user-friendly tool for health professionals and, as a free application, does not represent any additional cost [23].

Compared with the studies identified in the literature it is expected that data mining techniques could induce predictions with greater accuracy than known traditional methods.

### Data comprehension

The study was performed at the Obstetrics Department of the Centro Hospitalar Tâmega e Sousa, E.P.E., North Portugal, during the period from February to March of 2011.

Healthy newborn infants with 35 or more weeks of gestation were included in the study. Thus, 4 cases without this requirement were excluded from the 231 in the initial sample.

All the data present in the newborn original paper-based record, collected by doctors and nurses, was transcribed into a Microsoft Access database previously implemented for this purpose.

The collected data included: mother and father information, siblings information, gestational information, delivery information, physical exam of the newborn and clinical information of the complete hospital stay. At total, 72 variables were collected and analyzed. The complete table with all the variables is presented in Additional file 1.

Also, transcutaneous bilirubin levels were measured from birth to hospital discharge with maximum time intervals of 8 hours between measurements, using a noninvasive bilirubinometer, the JM-103 Jaundice Meter from Konica Minolta, following the manufacturer’s instructions. Once hyperbilirubinemia was diagnosed and phototherapy was provided, the further bilirubinometer measurements were not performed.

### Data preparation

A preliminary statistical analysis was carried out to increase knowledge about the dataset.

During this statistical analysis we performed the data preparation that included elimination, integration, recoding and calculation of variables. All these transformations are presented in detail in Additional file 1.

Eliminated variables – only variables with all missing values have been eliminated, that is, those variables whose information was not collected by doctors and nurses.

Integrated variables – in the newborn paper record, different variables collected repeated information, therefore we integrated the information of these variables into new ones.

Recoded variables – to facilitate the statistical analysis, some variables were also recoded (transformed).

Calculated variables – some variables, such as the dates of admission and discharge, were used to calculate new variables (e.g., length of hospital stay).

After the preparation of data, 60 out of 72 variables remained, plus the transcutaneous bilirubin levels. The final dataset was converted to be modeled using WEKA.

### Modeling

To perform data modeling, different classification algorithms, often applied in medical datasets and implemented in WEKA, were chosen: *J48* (implementation of the C4.5 algorithm, for generating pruned or unpruned decision trees), *simple CART* (a decision tree learner implementing minimal cost complexity pruning), *naïve Bayes* (a Naïve Bayes classifier using estimator classes), *multilayer perceptron* (a classifier that uses backpropagation to classify instances), *SMO* (implements John Platt’s sequential minimal optimization algorithm for training a support vector classifier) and *simple logistic* (classifier for building linear logistic regression models). Other similar methods were also used but without better results and, therefore, are not reported in this study.

The tests were performed using internal cross validation 10-folds. The internal cross-validation is used to determine how the quality of a learning algorithm will be affected in separate sets of data.The average performance on the test set provides an estimate of the performance of the classifier built from the entire data set [20,24,25].

xAll classification algorithms were tested for different subsets of variables and compared in terms of accuracy, sensitivity and specificity. For all subsets, we established a sensitivity of 90% and calculated the respective specificity due to the importance of high sensitivity values in medical decision. Standard error for all AUC measurements was estimated using the method proposed by Hanley and McNeil [26].

The different subsets corresponded to three different moments. First we used only risk factors that were obtained immediately after the newborns birth: Mother age; Father age; Head circumference; Mother pathologies; Mother usual medication; Gestational age; Physical exam report; Type of delivery; Newborn blood group (Rh); Newborn blood group (ABO) and Mother blood group (ABO).

Then, we also tested the algorithms with the TcB levels, without other risk factors, obtained until 24 hours of life of the newborn.

Finally, we tested the combination of the risk factors and the TcB levels at 24 hours of life of the newborn.

An approval was obtained from the Ethics Committee of the Centro Hospitalar Tâmega e Sousa, EPE, having the reference number 0568/2011.

## Results

From the total of 227 newborn infants included into the study, 35 cases (15.4%) were diagnosed with hyperbilirubinemia and treated with phototherapy, the predictive outcome of the study.

The 35 newborn infants treated with phototherapy initiated treatment with a median age of 45.5 hours and early jaundice, detected before the newborn completes 24 hours of life, was present in 4 cases (11.4%).

In the first step, applying the algorithms to the clinical risk factors, a higher accuracy was obtained with Bayes net algorithm (AUC=0.74), followed by *naïve bayes* and *simple logistic* (AUC=0.72).

Using only the TcB levels obtained before 24 hours of life of the newborn, higher accuracy was obtained with the *multilayer perceptron*, the WEKA artificial neural network algorithm (AUC=0.84) followed by *naïve Bayes* (AUC=0.82) and *simple logistic* (AUC=0.80).

When combining clinical risk factors with TcB, at 24 hours of life of the newborn, higher accuracy was obtained with *simple logistic* algorithm (AUC=0.89) followed by *naïve Bayes* (AUC= 0.88) and *Bayes net* (AUC=0.87).

In all algorithms, except the *multilayer perceptron*, the combination of clinical risk factors with TcB levels allowed to improve the accuracy of prediction when compared with TcB or clinical risk factors alone.

Table 2 presents the results from the comparison of the different algorithms applied to data subsets.

**Table 2:** Comparison of the application of different algorithms to data subsets in terms of accuracy and specificity (for sensitivity of 90%)

**Algorithms**	**Subsets**								
	**CRF**			**TcB at 24 h**			**TcB and CRF at 24 h**		
	**AUC**	**95% CI**	**SPE**	**AUC**	**95% CI**	**SPE**	**AUC**	**95% CI**	**SPE**
**J48**	0.47	(0.42-0.52)	0.09	0.79	(0.74-0.84)	0.43	0.75	(0.70-0.80)	0.33
**Simple Cart**	0.46	(0.41-0.51)	0.10	0.76	(0.71-0.81)	0.42	0.77	(0.72-0.82)	0.41
**Naive Bayes**	0.72	(0.67-0.77)	0.38	0.82	(0.77-0.87)	0.54	0.88	(0.84-0.92)	0.56
**Bayes Net**	0.74	(0.69-0.79)	0.42	0.73	(0.68-0.78)	0.35	0.87	(0.83-0.91)	0.60
**MP**	0.70	(0.65-0.75)	0.35	0.84	(0.80-0.88)	0.53	0.81	(0.76-0.86)	0.50
**SMO**	0.53	(0.48-0.58)	0.15	0.50	(0.45-0.55)	0.12	0.72	(0.67-0.77)	0.54
**Simple Logistic**	0.72	(0.67-0.77)	0.39	0.80	(0.75-0.85)	0.41	0.89	(0.85-0.93)	0.56

MP – Multilayer Perceptron; SMO – Sequential Minimal Optimization; AUC – Area under the receiving-operator characteristic curve; CI – Confidence interval; SPE – Specificity; CRF – Clinical Risk Factors; TcB – Transcutaneous bilirubin.

## Discussion

When compared with the traditional methods, the prediction with the application of data mining techniques offered interesting results.

Comparing with the literature, and specifically with a study from Chou et al. [14] which also sought to provide information for the indication for phototherapy, this study shows improved results with an AUC of 0.74, compared to the 0.69 presented in that study, although the differences are not statistically significant (the confidence intervals overlap). But, when compared with other studies, particularly a study by Newman, et al. [16] which seeks to predict bilirubin levels above 25 mg/dl, and safeguarding the differences, our study presented falls short of the 0.83 presented.

Despite not presenting so good results, decision trees models, generated using for instance J48 or Simple Cart, have the advantage of being more easily interpretable, especially when compared with closed models, usually called black box models, such as Artificial Neural Networks. This advantage makes the first to be more easily accepted by the medical community [24,27].

Regarding the bilirubin assessment, the identified studies seek to predict the risk of subsequent hyperbilirubinemia using predischarge TSB values. In the present study we used the first day TcB level, to predict the need for phototherapy.

With the application of the multilayer perceptron algorithm, we obtained a slightly higher accuracy than Keren & Bhutani [17], with an AUC of 0.84, compared with AUC of 0.83, however, this difference is not statistically significant because our result falls in the confidence interval presented in their study.

However, in practice, because it presents better accuracy results, the pediatricians base their assessment in the combination of clinical risk factors with the bilirubin levels presented by the newborns. This is also the methodology supported by the international guidelines from AAP and NICE.

Applied to our dataset, the simple logistic algorithm returned better results than those presented by Newman, et at [16]: we obtained an accuracy of 0.89 compared to 0.86 in their study. Once more, this difference is not statistically significant, since the confidence intervals overlap.

In addition to the comparison of accuracy it is also important to make an interpretation of the generated models and compare them with clinical rules of thumb, that is, what actually prevails in practice.

Thus, taking as an example the results obtained with the *simple logistic* algorithm, which is one of the best performing models in all feature subsets, we found that, when applied to the subset containing risk factors and transcutaneous bilirubin levels, the variables with higher influence are, in descending order: *TcB* in the range between 8 to 16 hours, *TcB* in the range 16 to 24 hours, *gestational age* and *newborn blood group* (*ABO*).

It is interesting to note that, with regard to TcB levels, the range 8 to 16 hours has greater influence than the subsequent interval, between 16 to 24 hours. It is also important to underline that the first interval between 0 and 8 hours of the newborn life is not part of the generated model. This may be due to the low register of values in the first interval of 8 hours. However, it also reflects the importance of assessment and registration of TcB as early as possible, as supported by several studies.

Concerning risk factors, the algorithm used only the variables *gestational age* and *newborn blood group* (*ABO*) for building the model when, in daily practice, the presence of any risk factor guidelines described by the presence, for example, of cephalhematomas or previous sibling with phototherapy, are considered as an equal increase in risk for subsequent hyperbilirubinemia.

These results are similar to studies that indicate the gestational age as the most determinant variable in the prognosis of neonatal jaundice [28]. However, the newborn *blood group (ABO)* acquires a prominent position in the generated model, since it can be related to the cases of jaundice derived from blood incompatibility.

Resuming, preserving the differences, the application of data mining techniques allowed building high accuracy models, with results not lower than the traditional methods found in the literature.

As mentioned, the average age of newborns at the beginning of treatment is around 45.5 hours of life, a value very close to the possible time of hospital discharge. This makes us believe that an early correct assessment, which can be performed by the proposed methods – the application of data mining methods – can enable reducing effectively the time of admission, as well as prevent incorrect diagnoses for the same reason and reduce readmissions after hospital discharge.

### Limitations

The predictive outcome, hyperbilirubinemia, defined differently in the compared studies, may constitute an important bias factor.

The use of other data mining software’s besides WEKA, with different implementation of data mining algorithms, could eventually lead to different results.

A bigger sample could also improve the obtained results.

## Conclusion

Neonatal hyperbilirubinemia and kernicterus prevention is still one of the most defying problems that face pediatricians nowadays, even with the generalization of the AAP and NICE guidelines.

The main findings of this study showed that data mining techniques are important and valid approaches for the prediction of neonatal hyperbilirubinemia.

So, we recommend that new technologies, such as data mining, should be explored and utilized to support medical decision, contributing to improve diagnosis in neonatal jaundice.

## Competing interests

The authors declare that they have no competing interests.

## Authors’ contributions

All authors contributed equally in the research. All authors read and approved the final manuscript.

## Pre-publication history

The pre-publication history for this paper can be accessed here:

http://www.biomedcentral.com/1472-6947/12/143/prepub

## Supplementary Material

Additional file 1 Appendix. Click here for file

## References

[B1] Keren R Luan X Friedman S Saddlemire S Cnaan A Bhutani VK A comparison of alternative risk-assessment strategies for predicting significant neonatal hyperbilirubinemia in term and near-term infants Pediatrics 2008 121 1 e170 e179 10.1542/peds.2006-3499 18166536

[B2] Bhutani VK Vilms RJ Hamerman-Johnson L Universal Bilirubin screening for severe neonatal hyperbilirubinemia J Perinatol 2010 30 Suppl S6 S15 20877410 10.1038/jp.2010.98

[B3] Maisels MJ Screening and early postnatal management strategies to prevent hazardous hyperbilirubinemia in newborns of 35 or more weeks of gestation Semin Fetal Neonatal Med 2010 15 3 129 135 10.1016/j.siny.2009.10.004 20034861

[B4] NICE Detection and treatment of neonatal jaundice Lancet 2010 375 9729 1845 10.1016/S0140-6736(10)60852-5 20510996

[B5] Rennie J Burman-Roy S Murphy MS Neonatal jaundice: summary of NICE guidance BMJ 2010 340 c2409 10.1136/bmj.c2409 20484363

[B6] De Luca D NICE guidelines on neonatal jaundice: at risk of being too nice Lancet 2010 376 9743 771 20816543 10.1016/S0140-6736(10)61376-1

[B7] Smitherman H Stark AR Bhutani VK Early recognition of neonatal hyperbilirubinemia and its emergent management Semin Fetal Neonatal Med 2006 11 3 214 224 10.1016/j.siny.2006.02.002 16603425

[B8] Besser I Perry ZH Mesner O Zmora E Toker A Yield of recommended blood tests for neonates requiring phototherapy for hyperbilirubinemia Isr Med Assoc J 2010 12 4 220 224 20803881

[B9] Randev S Grover N Predicting neonatal hyperbilirubinemia using first day serum Bilirubin levels Indian J Pediatr 2010 77 2 147 150 10.1007/s12098-009-0335-3 20107937

[B10] Bhutani VK Johnson L Sivieri EM Predictive ability of a predischarge hour-specific serum Bilirubin for subsequent significant hyperbilirubinemia in healthy term and near-term newborns Pediatrics 1999 103 1 6 14 10.1542/peds.103.1.6 9917432

[B11] AAP Management of hyperbilirubinemia in the newborn infant 35 or more weeks of gestation Pediatrics 2004 114 1 297 316 15231951 10.1542/peds.114.1.297

[B12] Manning D Todd P Maxwell M Jane Platt M Prospective surveillance study of severe hyperbilirubinaemia in the newborn in the UK and Ireland Arch Dis Child Fetal Neonatal Ed 2007 92 5 F342 F346 10.1136/adc.2006.105361 17074786 PMC2675352

[B13] Burke BL Robbins JM Bird TM Hobbs CA Nesmith C Tilford JM Trends in hospitalizations for neonatal jaundice and kernicterus in the united states, 1988–2005 Pediatrics 2009 123 2 523 532 10.1542/peds.2007-2915 19171618

[B14] Chou SC Palmer RH Ezhuthachan S Newman C Pradell-Boyd B Maisels MJ Testa MA Management of hyperbilirubinemia in newborns: measuring performance by using a benchmarking model Pediatrics 2003 112 6 Pt 1 1264 1273 14654595 10.1542/peds.112.6.1264

[B15] Bernaldo AJ Segre CA Bilirubin dosage in cord blood: could it predict neonatal hyperbilirubinemia? Sao Paulo Med J 2004 122 3 99 103 10.1590/S1516-31802004000300005 15448807 PMC11126191

[B16] Newman TB Liljestrand P Escobar GJ Combining clinical risk factors with serum Bilirubin levels to predict hyperbilirubinemia in newborns Arch Pediatr Adolesc Med 2005 159 2 113 119 10.1001/archpedi.159.2.113 15699303

[B17] Keren R Bhutani VK Predischarge risk assessment for severe neonatal hyperbilirubinemia NeoReviews 2007 8 e68 e76 10.1542/neo.8-2-e68

[B18] Malucelli A Stein Junior A Bastos L Carvalho D Cubas MR Paraiso EC Classification of risk micro-areas using data mining Rev Saude Publica 2010 44 2 292 300 10.1590/S0034-89102010000200009 20339628

[B19] Worachartcheewan A Nantasenamat C Isarankura-Na-Ayudhya C Pidetcha P Prachayasittikul V Identification of metabolic syndrome using decision tree analysis Diabetes Res Clin Pract 2010 90 1 e15 e18 10.1016/j.diabres.2010.06.009 20619912

[B20] Chen HY Chuang CH Yang YJ Wu TP Exploring the risk factors of preterm birth using data mining Expert Syst Appl 2011 38 5 5384 5387 10.1016/j.eswa.2010.10.017

[B21] Delen D Walker G Kadam A Predicting breast cancer survivability: a comparison of three data mining methods Artif Intell Med 2005 34 2 113 127 10.1016/j.artmed.2004.07.002 15894176

[B22] Shearer C The CRISP-DM model: the New blueprint for data mining Journal of Data WareHousing 2000 5 13 22

[B23] Vianna RC Moro CM Moyses SJ Carvalho D Nievola JC Data mining and characteristics of infant mortality Cad Saude Publica 2010 26 3 535 542 10.1590/S0102-311X2010000300011 20464072

[B24] Delen D Oztekin A Kong ZJ A machine learning-based approach to prognostic analysis of thoracic transplantations Artif Intell Med 2010 49 1 33 42 10.1016/j.artmed.2010.01.002 20153956

[B25] Kuzniewicz MW Escobar GJ Wi S Liljestrand P McCulloch C Newman TB Risk factors for severe hyperbilirubinemia among infants with borderline Bilirubin levels: a nested case–control study J Pediatr 2008 153 2 234 240 10.1016/j.jpeds.2008.01.028 18534217 PMC3142930

[B26] McNeil BJ Hanley JA Funkenstein HH Wallman J Paired receiver operating characteristic curves and the effect of history on radiographic interpretation. CT of the head as a case study Radiology 1983 149 1 75 77 6611955 10.1148/radiology.149.1.6611955

[B27] Oztekin A Delen D Kong ZJ Predicting the graft survival for heart-lung transplantation patients: an integrated data mining methodology Int J Med Inform 2009 78 12 e84 e96 10.1016/j.ijmedinf.2009.04.007 19497782

[B28] Goncalves A Costa S Lopes A Rocha G Guedes MB Centeno MJ Silva J Silva MG Severo M Guimaraes H Prospective validation of a novel strategy for assessing risk of significant hyperbilirubinemia Pediatrics 2011 127 1 e126 e131 10.1542/peds.2009-2771 21149428

