# Supplementary material for: Applying data mining techniques to improve diagnosis in neonatal jaundice
Source: BMC Med Inform Decis Mak. 2012 Dec 7;12:143. doi: 10.1186/1472-6947-12-143 (PMC3557145; doi:10.1186/1472-6947-12-143)
Supplement: Additional file 1 — Appendix. [file 1472-6947-12-143-S1.docx]

Appendix A Variables initially collected in the study

| **Parental information** | |
| --- | --- |
| Mother blood group | Father blood group |
| Mother age | Father age |
| Mother race | Father race |
| Mother smoking habits | Father pathologies |
| Mother alcohol consumption | Number of siblings |
| Mother drug dependence | Siblings with jaundice |
| Mother pathologies | Siblings treated with phototherapy |
| Mother usual medication | Sibling pathologies |
| **Previous pregnancies** |  |
| Number of pregnancies | Stillborn |
| Number of births | Voluntary interruptions of pregnancy |
| Previous abortions | Medical interruptions of pregnancy |
| **Current pregnancy** |  |
| Number of appointments | Hepatitis B screening |
| First appointment date | Syphilis screening |
| Appointments place | Toxoplasmosis screening |
| Term appointment in CHTS | Rubella screening |
| Last menstrual period | Cytomegalovirus screening |
| Gestational age by last menstrual period | Other screenings |
| Gestational age by ultrasound | Ultrasound results |
| Indirect Coombs test | Biochemical screening |
| Anti-D immunoglobulin | Amniocentesis |
| Pregnancy pathologies | Group B streptococcus screening |
| Pregnancy medication | Group B streptococcus prophylaxis |
| HIV screening |  |
| **Delivery information** |  |
| Date and time of delivery | Analgesia |
| Type of delivery | 1st minute Apgar score |
| Amniotic fluid characteristics | 5th minute Apgar score |
| Rupture of amniotic membranes | 10th minute Apgar score |
| Antibiotics administered | Need for resuscitation |
| Other medication administered |  |
| **Newborn Clinical data** |  |
| Gender | Coombs test |
| Birth weight | Cephalhematomas or significant bruising |
| Length | Report of physical examination |
| Head circumference | Type of feeding |
| Umbilical cord vessels | Weight at discharge |
| Blood group |  |
| Transcutaneous bilirubin levels | |
| Blood bilirubin levels | |
| **Phototherapy** |  |
| Date and starting time | Date and stopping time |
| **Clinical discharge** |  |
| Date and time of clinical discharge |  |

Appendix B data preparation process

| **Eliminated variables** | | |
| --- | --- | --- |
| Mother drug dependence | Hepatitis B screening | |
| Stillborn | Syphilis screening | |
| Voluntary interruptions of pregnancy | Other screenings | |
| Indirect Coombs test | Umbilical cord vessels | |
| HIV screening | Number of pregnancies | |
| Medical interruptions of pregnancy |  | |
| **Integrated variables** | | |
| Number of appointments | → | Monitoring of pregnancy |
| First appointment date |  |  |
| Last menstrual period | → | Gestational age |
| Gestational age by last menstrual period |  |  |
| Gestational age by ultrasound |  |  |
| **Recoded variables** | | |
| Mother blood group | → | Mother blood group (ABO) |
|  | → | Mother blood group (Rh) |
| Father blood group | → | Father blood group (ABO) |
|  | → | Father blood group (Rh) |
| Newborn blood group | → | Newborn blood group (ABO) |
|  | → | Newborn blood group (Rh) |
| **Calculated variables** | | |
| Weight loss (Weight at discharge - Birth weight) | | |
| Hospital length of stay (Date and time of clinical discharge - Date and time of delivery) | | |
| Duration of phototherapy (Date and stopping time - Date and starting time) | | |
| Early jaundice (Phototherapy date and starting time - Date and time of delivery) | | |
